# Bartonella-Associated Endocarditis with Severe Active Crescentic Glomerulonephritis and Acute Renal Failure

**DOI:** 10.1155/2021/9951264

**Published:** 2021-05-29

**Authors:** Ramanath Dukkipati, Benjamin Lawson, Cynthia C. Nast, Anuja Shah

**Affiliations:** ^1^Harbor-UCLA Medical Center, Torrance, California, USA; ^2^Lundquist Research Institute, Torrance, California, USA; ^3^UCLA School of Medicine, Westwood, California, USA; ^4^Cedars-Sinai Medical Center, Los Angeles, California, USA

## Abstract

We report a case of severe acute kidney failure due to crescentic glomerulonephritis who presented initially with culture-negative endocarditis with vegetations on the aortic valve. Anti-nuclear and anti-phospholipid antibodies were positive with initially negative anti-neutrophil cytoplasmic antibodies (ANCAs). Kidney biopsy revealed severe acute crescentic glomerulonephritis with mesangial immune complex deposition. PR3-ANCA subsequently become positive, and the patient developed worsening kidney failure requiring hemodialysis. This case illustrates that Bartonella can present as culture-negative endocarditis with severe crescentic glomerulonephritis with positive PR-3 ANCAs and can mimic ANCA-associated crescentic glomerulonephritis.

## 1. Background

Infective endocarditis- (IE-) associated glomerulonephritis (GN) has been reported to present commonly with acute kidney injury (AKI) and tricuspid valve infection due to Staphylococcus. Over half of the patients usually have an underlying cardiac abnormality. The most common bacteria isolated have been reported to be Staphylococcus and Streptococcus. Glomerulonephritis that is associated with infection is diverse, but the most common pattern of glomerular injury in IE-associated glomerulonephritis is crescentic and necrotizing glomerulonephritis [1, 2]. There are only few reported cases of *Bartonella henselae-*associated IE presenting with severe renal failure due to necrotizing and crescentic glomerulonephritis [3, 4].

We report a case of a 70-year-old female who presented with severe renal failure due to acute crescentic glomerulonephritis and Bartonella-associated vegetation and insufficiency of the aortic valve. At presentation, she tested negative for PR-3 ANCA and MPO-ANCA but later during the course of hospitalization became positive for PR-3 ANCA. She became dialysis dependent and was treated with rifampin and doxycycline.

## 2. Case Report

A 70-year-old African-American female presented with a one month history of failure to thrive, decreased oral intake, dizziness, and weakness. She complained of having an unintentional weight loss of 15 pounds over the past month due to decreased appetite and episodes of non-bloody diarrhea over the past two weeks. She did not complain of fevers or joint pains and had no family history of kidney disease. She reported tobacco use for twenty years until one month prior to this presentation. The past medical history was significant for hypertension for which she was taking hydralazine and hydrochlorothiazide and for hypothyroidism which was treated with levothyroxine.

Physical examination showed no rash, joint swelling, or leg edema. During hospitalization, her temperatures did not exceed 100° Fahrenheit. Results of the laboratory investigations on the day of admission to the hospital are shown in Table 1. HIV, hepatitis C, and hepatitis B surface antigen were negative; anti-nuclear antibody, *β*2 microglobulin IgM, and anti-cardiolipin IgM studies were positive, complement 3 (C3) was low, and complement 4 (C4) was normal. Blood cultures drawn on the day of admission were negative. She was negative for peri-nuclear anti-neutrophil cytoplasmic auto antibodies (P-ANCAs) and negative for cytoplasmic anti-neutrophil cytoplasmic auto antibodies (C-ANCAs) by indirect immunofluorescence test. The patient was started on vancomycin and tazobactam/piperacillin initially while awaiting blood culture results, which came back as negative 48 hours after admission.

**Figure 1:**
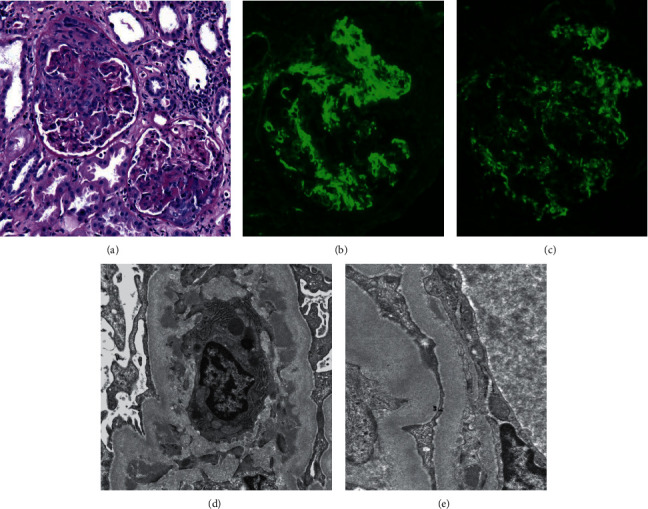
Kidney biopsy images. (a) Glomeruli with cellular crescents, one of which also shows segmental necrosis (arrow). Periodic-acid Schiff. (b) Strong granular mesangial staining for C3 by immunofluorescence. (c) Weak granular mesangial staining for IgG by immunofluorescence. (d) Electron microscopy showing mesangial deposits (arrows) and (e) small subendothelial deposit (arrow).

A kidney biopsy was done on day 3 of hospitalization (Figure 1). There were 32 glomeruli, 10 of which (31%) were globally sclerotic. There were one necrotizing and 10 cellular and fibro cellular crescents in 11 patent glomeruli while four glomeruli had fibrotic crescents. No mesangial hypercellularity, large subendothelial deposits, or fibrin thrombi were present nor was there endocapillary hypercellularity other than that associated with crescents. Tubular cells showed degeneration, and the interstitium was edematous with a mild diffuse mononuclear inflammatory infiltrate. There was very mild tubular atrophy with interstitial fibrosis. Arteries and arterioles had no inflammation, necrosis, thrombi, or micro-angiopathic features. Immunofluorescence showed weak IgG (1+) staining and IgA (trace) staining, moderate C1q (1‒2+) and IgM (2+) staining, and strong C3 (2‒3+) granular staining in mesangial regions and very segmentally along capillary walls. Electron dense deposits were in mesangial regions, and very segmentally small deposits were in subendothelial locations; there were no subepithelial deposits. Podocyte foot processes were 50% effaced, and there were no tubulo-reticular inclusions. She was diagnosed with immune complex-mediated glomerulonephritis with active crescentic glomerulonephritis and associated acute tubulointerstitial injury.

**Figure 2:**
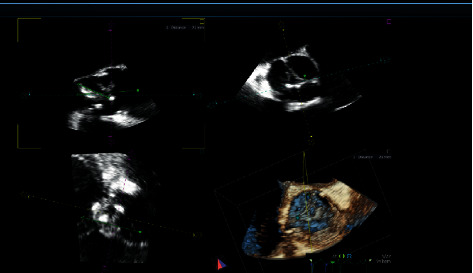
Echocardiogram images showing vegetations (densities) on the aortic valve with severe aortic regurgitation (arrow shows the vegetations).

The patient continued to have a normal temperature during hospitalization. PR-3 ANCAs became positive (4.5 where less than 1 is negative) on day 10 of hospitalization while the MPO-ANCAs remained negative. A murmur heard on physical examination prompted transthoracic echocardiogram, which revealed linear densities on the aortic valve (Figure 2). A trans-esophageal echocardiogram confirmed the presence of vegetations on the aortic valve with severe aortic regurgitation (Figure 2). An infectious disease consultation was obtained, and additional tests were ordered for culture-negative endocarditis. Two weeks after the initial presentation, reverse-transcriptase polymerase chain reaction (PCR) results from the infectious disease evaluation were positive for *Bartonella henselae*. She was started on doxycycline and rifampicin, and tazobactam/piperacillin and vancomycin were discontinued. Anti-TPO (thyroperoxidase) antibodies were not checked. The serum creatinine remained elevated (admission serum creatinine was 7.81 mg/dl) with oliguria, and hemodialysis was started via a right internal jugular tunneled dialysis catheter 4 days after hospitalization. Cardiac surgery for aortic valve replacement was scheduled to follow 6 weeks of antibiotic therapy. Her renal function did not recover, and she remained dependent on dialysis. Microbial cell-free DNA testing was performed on the plasma and was positive for *Bartonella henselae* at 14,2444 molecules per microliter, and the reference range for normal is less than 10.

## 3. Discussion

We describe a biopsy-proven case of severe acute crescentic glomerulonephritis with acute renal failure requiring hemodialysis in a patient with aortic valve endocarditis due to *Bartonella henselae*. *B*. *henselae* is endemic worldwide and was a common infection during World War I, where it was termed “trench fever” [5]. It has been detected in a wide range of animals such as bats, cats, rodents, and whales, which serve as reservoirs for potential zoonotic infection, and is a re-emerging infectious disease in humans [5]. Transmission to humans has been linked to cats, prompting the common term cat scratch disease. This patient likely had this route of transmission as she owned a cat. Approximately 90–95% of native valve endocarditis cases are associated with positive blood cultures; however, Bartonella does not grow in routine blood culture [6]. Other bacteria that grow poorly or not at all in culture-negative endocarditis include *Coxiella burnetti*, Legionella, and *Tropheryma whipplei* [6]. Therefore, *Bartonella henselae*, along with other above-listed infectious agents, should be considered as potential pathogenic organisms in what appears to be culture-negative endocarditis at presentation. The patient presented in this report is unique compared to previous reports in that there is a link to a zoonotic exposure and the crescentic glomerulonephritis was severe leading to acute renal failure requiring dialysis. Our patient likely had degeneration of the aortic valve which was not visible on the echocardiogram.

Endocarditis-associated kidney disease may present with a rapidly progressive picture and biopsy findings of necrotizing and crescentic glomerulonephritis in up to 65% [1, 7]. Overall, endocarditis is associated with a positive ANCA, typically P-ANCA, in up to 33% but in 64% when associated with *Bartonella henselae* [1–3]. It is critical to identify endocarditis, particularly that associated with Bartonella, as the underlying pathogenetic process in such cases to avoid immunosuppression, which has not been shown to be effective in crescentic GN secondary to endocarditis [2]. In the present case, the findings of active crescents with mesangial deposits dominant for C3 and a lack of tubulo-reticular inclusions suggested an etiology other than ANCA-associated vasculitis or lupus nephritis as the cause of the glomerular disease.  Table 2 summarizes what we think are key teaching points in this case. Anti-phospholipid antibodies which were positive in this case led us initially to consider thrombotic microangiopathy as a possible cause of acute renal failure and Libman–Sacks endocarditis, but retrospectively, these were positive likely secondary to her infection similar to the presence of these antibodies in infections. There was no evidence of thrombotic angiopathy seen in the kidney biopsy. Bartonella can be transmitted by cats and therefore is called “cat scratch disease.” Our patient did live in the vicinity of feral cats. However, she did not have a pet cat herself and did not confess to having been scratched by a cat. Therefore, it is unclear how she acquired Bartonella infection.

In summary, this case highlights the importance of a detailed workup for infectious disease even if routine initial blood cultures are negative in cases of crescentic glomerulonephritis before immunosuppression is initiated. Re-emergence of zoonotic infectious pathogens such as Bartonella should be appreciated as an important cause of culture-negative endocarditis and crescentic glomerulonephritis.

## Figures and Tables

**Table 1:** Results of laboratory investigations.

	Day of admission
Proteinuria urine protein/creatinine ratio	2.21
Hematuria (RBC per HPF)	11–25
Creatinine (mg/dL) (normal: 0.8–1.0 mg/dL)	7.81
eGFR (ml/min/1.73 m^2^)	6
Albumin (g/L) (4.0–4.5 g/L)	2.4
Complement factor 3 (g/l) (normal: 79–152)	46
Complement factor 4 (g/l) (normal: 16–38)	25
Anti-nuclear antibody (normal: less than 1 : 40) speckled pattern	1 : 80
Anti-double stranded DNA	Negative
Anti-neutrophil cytoplasmic antibody (ANCA) MPO	Negative
ANCA PR 3	Negative
Anti-phospholipid antibodies (beta-2-microglobulin IgM and cardiolipin IgM)	Positive

**Table 2:** Key teaching points.

(1) *Bartonella henselae* should be checked in cases of culture-negative endocarditis who present with renal failure. Bartonella avoids detection by routine blood cultures. Detection requires specific growth medium and extended incubation periods.
(2) PR-3 ANCA may be positive in *Bartonella henselae* endocarditis with crescentic glomerulonephritis. A complete infectious workup should be done before immunosuppression is initiated in patients with crescentic glomerulonephritis.
(3) In patients with infective endocarditis, the predominant pattern of glomerular injury is crescentic glomerulonephritis.
(4) Since there are no bactericidal antibiotics available to treat *Bartonella henselae*, the antigen may persist even after antibiotic administration, potentially allowing a continued immune response with immune complex formation for days to weeks after antibiotic therapy initiation.
